# High sensitivity of tropical forest birds to deforestation at lower altitudes

**DOI:** 10.1002/ecy.3867

**Published:** 2022-11-14

**Authors:** Simon C. Mills, Jacob B. Socolar, Felicity A. Edwards, Edicson Parra, Diego E. Martínez‐Revelo, Jose Manuel Ochoa Quintero, Torbjørn Haugaasen, Robert P. Freckleton, Jos Barlow, David P. Edwards

**Affiliations:** ^1^ Ecology and Evolutionary Biology, School of Biosciences University of Sheffield Sheffield UK; ^2^ Faculty of Environmental Sciences and Natural Resource Management Norwegian University of Life Sciences Ås Norway; ^3^ Cornell Lab of Ornithology Cornell University Ithaca New York USA; ^4^ RSPB Centre for Conservation Science, RSPB Cambridge UK; ^5^ Asociación GAICA Pasto Colombia; ^6^ Instituto de Investigación de Recursos Biológicos Alexander von Humboldt Bogotá Colombia; ^7^ Lancaster Environment Centre Lancaster University Lancaster UK

**Keywords:** avian community, elevational gradients, forest conversion, montane tropics, tropical conservation

## Abstract

Habitat conversion is a major driver of tropical biodiversity loss, but its effects are poorly understood in montane environments. While community‐level responses to habitat loss display strong elevational dependencies, it is unclear whether these arise via elevational turnover in community composition and interspecific differences in sensitivity or elevational variation in environmental conditions and proximity to thermal thresholds. Here we assess the relative importance of inter‐ and intraspecific variation across the elevational gradient by quantifying how 243 forest‐dependent bird species vary in sensitivity to landscape‐scale forest loss across a 3000‐m elevational gradient in the Colombian Andes. We find that species that live at lower elevations are strongly affected by loss of forest in the nearby landscape, while those at higher elevations appear relatively unperturbed, an effect that is independent of phylogeny. Conversely, we find limited evidence of intraspecific elevational gradients in sensitivity, with populations displaying similar sensitivities to forest loss, regardless of where they exist in a species' elevational range. Gradients in biodiversity response to habitat loss thus appear to arise via interspecific gradients in sensitivity rather than proximity to climatically limiting conditions.

## INTRODUCTION

Tropical mountains are hyperdiverse regions of key conservation concern (Myers et al., 2000; Quintero & Jetz, 2018). They harbor a disproportionate fraction of the world's biodiversity, support large numbers of endemic and small‐range species, and provide important refugia for tropical species as the climate warms (Freeman et al., 2018). They are also considerably threatened by habitat loss and degradation, with many tropical mountain ranges displaying large human footprints (Elsen et al., 2020) and retaining just a small fraction of their natural habitat (e.g., just 31% of original forest remains in the northern Andes; Etter et al., 2006). Despite their global importance (Mittermeier et al., 2011; Myers et al., 2000), the effects of habitat loss and land‐use change on tropical montane biodiversity remain poorly understood (Elsen et al., 2020; O'Dea & Whittaker, 2007; Soh et al., 2019).

Community response to landscape modification was previously found to vary markedly with elevation, with highly modified landscapes displaying reduced species richness relative to unmodified landscapes toward lower elevations (~1000 m above sea level [MASL]) but little difference above 2000 MASL (Peters et al., 2019). Community‐level analyses of high‐elevation avifaunas suggest that these are relatively robust to habitat loss and degradation (Fjeldså, 1993; Marsden et al., 2006; O'Dea & Whittaker, 2007), particularly when contrasted with findings from the lowland tropics (e.g., Socolar & Wilcove, 2019). Two broad hypotheses are given to explain this contrast in the response of low‐ and high‐elevation communities. First, species assemblages that are found at different elevations may systematically vary in their response to loss of natural habitat, with typical low‐elevation species tending to be more sensitive to deforestation than those at higher elevations (Betts et al., 2019). Secondly, sensitivity may vary with proximity to climatic thresholds, with forest loss having relatively greater impacts toward lower elevations where populations exist closer to their thermal physiological thresholds (Peters et al., 2019). However, as population‐ and species‐level responses across elevational gradients have yet to be empirically addressed, the underpinnings of elevational variation in sensitivity to habitat loss remains unclear.

Interspecific elevational gradients in sensitivity may arise for three main reasons. First, along the elevational gradient there are marked structural changes to forest, with higher‐elevation forest having a higher density of canopy gaps (Asner et al., 2014), reduced stature, loss of vertical stratification (Terborgh, 1977), and longer recovery times following disturbance (Guariguata, 1990). Toward the treeline, forest becomes naturally stunted and patchily distributed, eventually giving way to high‐elevation grassland. Many of the features associated with degradation (e.g., loss of structural complexity, fragmentation) are thus naturally present at higher elevations, conferring species in these landscapes with some degree of tolerance to disturbance (Betts et al., 2019). Second, low‐elevation communities contain a variety of specialized foraging guilds and microhabitat specialists that are absent at higher elevations and might be particularly sensitive to disturbance (Pigot et al., 2016). Lastly, because species and taxonomic groups are highly nonrandomly assorted along the elevational gradient (Terborgh, 1977), if taxonomic groups associated with lower elevations tend to be more sensitive than those found at higher elevations, then gradients in sensitivity could also emerge via phylogenetically structured responses to habitat loss.

Intraspecific gradients in sensitivity are expected to occur due to interactions between proximity to climatic thresholds and habitat quality and connectivity. Populations that exist close to climatic thresholds are more strongly perturbed by climatic variation (Gerst et al., 2011) and likely to be ephemeral populations at heightened risk of extirpation (Feldman et al., 2015). Habitat features that modify a species' capacity to recolonize following local extirpation (e.g., forest connectivity) or help species avoid critical climatic episodes (e.g., forest structural complexity, access to microrefugia) are expected to be relatively more important in supporting long‐term population persistence in climatically marginal regions than in other portions of a species' range (González‐del‐Pliego et al., 2020; Oliver et al., 2015; Suggitt et al., 2018). Forest‐dwelling bird sensitivities to forest loss are expected to be particularly acute at warm range limits (i.e., lower elevations) since deforested landscapes tend to be warmer and drier than contiguous forest (Frishkoff et al., 2016; Karp et al., 2018).

**FIGURE 1:**
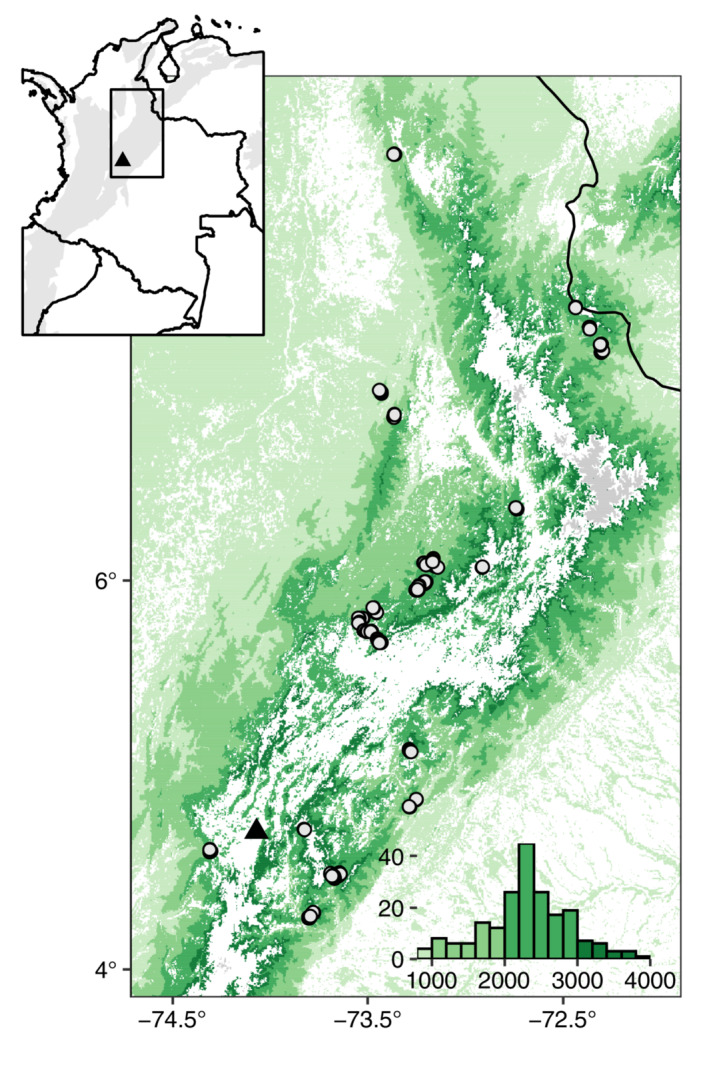
Map of fieldwork locations in eastern cordillera of Colombia. Inset panel displays study area within Colombia (black box), with montane regions (gray) and location of Bogota (triangle) overlaid. Main figure displays point‐count locations (circles) and forest cover (shaded by elevational position, green) (Hansen et al., 2013). Areas above 4000 m above sea level are shaded gray. Inset histogram displays elevations of points surveyed, following the same color scheme as in the main figure. Study region measures 47,000 km^2^ (convex hull).

In this study, we assessed how 243 forest birds responded to landscape‐scale forest loss across a large elevational gradient (880–3900 MASL) in the Eastern Andes of Colombia (Figure 1), examining both interspecific and intraspecific elevational variation while also accounting for phylogeny. The study region holds some of the highest levels of avian species richness and endemism globally (Mittermeier et al., 2011) but is heavily populated and deforested (69% deforestation in the Colombian Andes; Etter et al., 2006). We focused on birds for their exceptionally well‐quantified elevational ranges and habitat associations and restricted our analyses to species with some degree of forest dependency so as to use remotely sensed forest cover to measure habitat availability in the landscape. Specifically, we ask, first, whether forest‐dependent species found at different elevations vary in their sensitivity to loss of forest in the local landscape and, second, whether populations present toward species' lower and upper elevational range limits vary in their sensitivity to loss of forest in the local landscape.

## MATERIAL AND METHODS

### Data set summary and survey design

Fieldwork was carried out across the Eastern Cordillera of the Colombian Andes over the course of three field seasons during 2018 (July–August) and 2019 (January–April and June–September). Replicated sampling was completed throughout the Cordillera at 200 points situated inside forest at elevations between 880 and 3880 MASL (794 point counts in total; Figure 1; Appendix S1: Figure S1). The sampling design followed Gilroy et al. (2014), with points arranged within clusters of three and 200 m between points. Where dictated by topography, impassable terrain, or forest configuration, points within clusters were more broadly spaced (95% of within‐cluster distances fell in the interval [190, 500]; median within‐cluster distance between points was 217 m). We defined sites as points within ~20 km of one another and that lack major biogeographic turnover beyond that captured by elevation. Within a site, clusters were placed a minimum of 500 m apart (93% of within‐site, between‐cluster distances exceeded 500 m), and the vast majority of between cluster distances substantially exceeded this (median distance 8680 m).

At each point, four counts were run over consecutive days (one per day) between 5:30 AM and 12:00 PM, with the visit order being varied across sampling days. In a small minority of cases (2%, *n* = 15), sampling was extended later into the afternoon due to heavy rain or wind that prevented point counts during the morning. Due to illness or logistical constraints, six points received three visits rather than four, a feature that we accommodated in our statistical model. Point counts were run for 10 min by experienced observers (SCM, JBS, DPE), with all birds that were either observed or heard within a 100‐m radius identified and noted. Point counts were recorded (Olympus LS10 or Tascam DR100 mk III with Sennheiser ME‐62 microphone) to allow for subsequent identification of any unknown vocalizations using online reference material and through cross‐comparison between observers (www.xeno-canto.org).

Across 794 point counts (i.e., point × visit combinations), we detected 243 forest species (see following discussion for definition) whose elevational range midpoints exceeded 880 m (McMullan et al., 2018; Quiñones, 2018). Across these species, we obtained 2892 species × point detections, and 4637 detections in total (i.e., species × visit detections). Point‐level detection frequency was highly variable across species but skewed toward low numbers of point‐level detections per species (e.g., 54% of species detected on three or fewer points; Appendix S1: Figure S2), with a minority of species observed on high numbers of points.

### Landscape variables, species ranges, habitat associations, and phylogeny

Forest cover in 2018 was obtained from Hansen et al. (2013). Cells were defined as forested based on a 50% canopy‐cover threshold, and from this percentage forest cover within 500 m of each point was calculated. Forest cover classification was checked against satellite imagery and field notes, which confirmed they were classifying forest cover well. Nonforested areas in our surveyed landscapes were largely dedicated to cattle grazing, and there were no instances (toward lower elevations) where plantations or agriculture was misclassified as forest under this approach. The total forest cover metric generated under alternative forest cover thresholds was highly correlated (Pearson's *r* of 0.98, 1.00, 1.00, between 25% and 30%, 40%, and 50% classifications, respectively), indicating alternative classifications were unlikely to affect the results presented here. Forest cover reflects several related changes to habitat quality in the local landscape, and landscapes with low levels of forest cover are also those with reduced core area (*r* = 0.90) and increased edginess (*r* = −0.87), as well as increased fragmentation and reduced connectivity (*r* = 0.86; Appendix S1: Figure S3).

Point elevations were obtained from the ALOS 30‐m‐resolution digital elevation model (Tadono et al., 2014), with elevation calculated as the average elevation within 50 m of a point. Forest cover and point elevations were both obtained via Google Earth Engine (Gorelick et al., 2017).

Forest birds were classified according to the Birdlife forest dependency database as those with either medium or high forest dependency. Birdlife defines medium‐dependency species as forest generalists that tend to breed in the forest interior but are also regularly found along forest margins and secondary forest and may actually be more commonly encountered in these habitats than in the interior. High‐dependency species are defined as species that are overall more conservative in their requirements, tending to only rarely be encountered in nonforest environments and typically occurring in the forest interior, though they may have some capacity to persist in secondary forest or small forest fragments (Buchanan et al., 2011). Nine migratory species were removed because their habitat requirements were more variable over the course of the year (varying habitat requirements between, e.g., passage, overwintering) and had temporally varying occupancy states within our sampling periods.

Colombia‐specific species' elevational range limits were obtained from two sources: Ayerbe Quiñones (2018) and McMullan et al. (2018). Both sources give very similar upper and lower elevational range limits (upper range limit *r* = 0.96; lower range limit *r* = 0.96; Appendix S1: Figure S4), and for each species a combined elevational range limit was generated by taking the average across each source. For 18 species that did not have an elevational range provided by McMullan et al., the Ayerbe Quiñones elevations were used directly. Species with elevational midpoints below 880 m (the lowest elevation sampled) could only ever be observed toward their upper range margins, and rangewide sensitivities for these species are therefore highly extrapolated and poorly constrained by data. We therefore opted to restrict analyses to species with elevational midpoints that lie within the range of sampled elevations (i.e., >880 m, *n* = 243). Lastly, we obtained 100 phylogenies with the Ericson backbone from birdtree.org and from these generated a single consensus phylogeny based on mean edge length.

### Statistical analysis

Three main statistical challenges must be accommodated in our modeling framework. First, despite substantial surveying effort, the diversity of tropical communities means that few species individually have a large number of detections (Appendix S1: Figure S2). It is important that we retain infrequently observed species in our analysis because it is likely that the most commonly observed species will also tend to be those least affected by land‐use change (Banks‐Leite et al., 2014), and inferences from frequently observed species alone are therefore likely to be unrepresentative of forest birds in general. Second, the comparative nature of the question and data set necessitates a model that attempts to account for the phylogenetic nonindependence of species due to their shared evolutionary histories (Freckleton & Rees, 2019). Lastly, because the data are highly structured, with points nested within clusters, within sites, and by observer (*n* = 3), we also need to account for spatial structure in the sampling design as well as the potential for observer‐level detection effects.

To satisfy these requirements, we employed a Bayesian hierarchical detection‐occupancy model (Devarajan et al., 2020; Dorazio & Royle, 2005; Korner‐Nievergelt et al., 2015) that directly modeled the contributions of detection and occupancy to the overall probability of observing a species while pooling variance between species, thereby enabling retention of infrequently encountered species. We included phylogenetic random effect terms that captured phylogenetic structure in intercept and habitat effects, a number of detection covariates such as observer and time‐of‐day effects, as well as site × species and cluster × species random effects on occupancy to account for the spatial nonindependence of clusters or biogeographic turnover between sites. Elevational ranges were modeled by first rescaling each point's elevation relative to each species' published range limits so that −1 was the species' lower range margin, 0 the range midpoint, and 1 the species' upper range margin. This scaling placed species ranges on a common scale, allowing us to fit shared range‐shape parameters across species. To minimize computational effort, rather than model large numbers of all‐0 detection histories that arise at elevations far outside a species' elevational distribution, for each species we clipped the data set to only include points within ±3 units of scaled elevation (note that the most extreme detection was at a scaled elevation of 2) (Socolar et al., 2021).

### Occupancy model

We are interested in how habitat loss at the landscape scale affects species occupancy in the remaining forests. This could vary based on (a) the position of a species on the elevational gradient, (b) the position of a species in the phylogeny, (c) a species' degree of forest dependency, and (d) the position of a population across a species' elevational range. To encompass these four sources of variation, we used the following model of occupancy probabilities:

$$\begin{array}{ll} \text{logit}\left(\psi_{i,k}\right)= & u_{\text{cluster}\left[i\right],k}+\gamma_{\text{site}\left[i\right],k}+\beta_{0,k,\mathrm{dep}\left[k\right]}\\ & +\beta_{1,k,\mathrm{dep}\left[k\right]}\text{scaled}\;{\text{elevation}}_{i,k}\\ & +\beta_{2,k,\mathrm{dep}\left[k\right]}{\text{scaled elevation}}_{i,k}^{2}\\ & +\beta_{3,\mathrm{dep}\left[k\right]}\text{elevational}\;{\text{midpoint}}_{k}\\ & +\beta_{4,k,\mathrm{dep}\left[k\right]}{\text{habitat}}_{i}\\ & +\beta_{5,\mathrm{dep}\left[k\right]}\text{elevational}\;{\text{midpoint}}_{k}\times {\text{habitat}}_{i}\\ & +\beta_{6,\mathrm{dep}\left[k\right],\text{rhalf}\left[i\right]}\text{scaled}\;{\text{elevation}}_{i,k}\times {\text{habitat}}_{i}\\ & +\beta_{7,\mathrm{dep}\left[k\right]}\times {\text{range breadth}}_{k}\\ & +\beta_{8,\mathrm{dep}\left[k\right]}\times {\text{range breadth}}_{k}\times {\text{habitat}}_{i}, \end{array}$$

where *i* indexes points and *k* indexes species. The probability of occupancy is able to vary between species × cluster combination, $u_{\text{cluster}\left[i\right],k}$, and site × species combination, $\gamma_{\text{site}\left[i\right],k}$ (i.e., each cluster × species and site × species combination can vary in its average occupancy). All subsequent fixed‐effect terms varied by species' forest dependency (dep[*k*]), so that each term was estimated independently for medium‐ and forest‐dependent species. Species' occupancy varied according to their intercept, $\beta_{0,k,\mathrm{dep}\left[k\right]}$, the position of a point in a species’ elevational range, $\beta_{1,k,\mathrm{dep}\left[k\right]}$ and $\beta_{2,k,\mathrm{dep}\left[k\right]}$, where a species exists on the elevational gradient, $\beta_{3,\mathrm{dep}\left[k\right]}$, and by the amount of forest within 500 m, $\beta_{4,k,\mathrm{dep}\left[k\right]}$. Variation in the effect of habitat loss depending on where a species is in the environmental gradient arose via the interaction term between species' elevational midpoints and habitat $\left(\beta_{5,\mathrm{dep}\left[k\right]}\right)$. To allow the effects of habitat loss to vary across a species' elevational range, a final term was introduced: $\beta_{6,\mathrm{dep}\left[k\right],\text{rhalf}\left[i\right]}$. For each forest‐dependency class, this corresponds to two coefficients, one for each range half, so that sensitivity to habitat loss was not constrained to have had the same effect at upper as at lower range margins. We additionally included two terms, $\beta_{7,\mathrm{dep}\left[k\right]}\times \text{range}\;{\text{breadth}}_{k}$, where range breadth is the breadth of the *k*th species’ elevational range, and $\beta_{8,\mathrm{dep}\left[k\right]}\times \text{range}\;{\text{breadth}}_{k}\times {\text{habitat}}_{i}$, the interaction between range breadth and habitat, in case species with narrower elevational ranges tend to be systematically more or less sensitive to loss of habitat (Socolar et al., 2021).

The cluster × species and site × species intercept terms and all species‐specific terms are fitted via random effects. The intercept and habitat effect terms are fitted with both species‐level and phylogenetic random effects, and the importance of phylogeny assessed as the proportion of these two variances owing to phylogeny $\left(\text{i.e.,},\lambda ,=,\frac{\sigma_{\text{phylo}}^{2}}{\sigma_{\text{phylo}}^{2}+\sigma_{\mathrm{spp}}^{2}}\right)$.

### Detection

Detection was allowed to vary between species, $\delta_{0,k}$, time of day (as a species‐specific effect, $\delta_{1,k}$), observer, $o_{i}$, and observer × species, $v_{i,k}$, combinations. Observer and observer × species terms were included to accommodate potential systematic differences between observers' familiarity with local avifauna:

$$\text{logit}\left(\theta_{i,j,k}\right)=o_{i}+v_{i,k}+\delta_{0,i}+\delta_{1,k}\times \mathrm{tim}e_{i,j},$$

where *j* indexes visit. Observations, $y_{i,j,k}$, are then related to these detection and occupancy components by

$$z_{i,k}∼\text{Bernoulli}\left(\psi_{i,k}\right),$$

$$y_{i,j,k}∼\text{Bernoulli}\left(z_{i,k}\times \theta_{i,j,k}\right).$$

The observer × site effect and all species‐specific parameters were fitted hierarchically, allowing for individual species to have elevational range associations that departed from the overall species average. The observer effect (which has just three levels) was fitted as a fixed effect. Priors on all parameters are weakly informative in that they avoid issues with densities that are strongly concentrated around 0 and 1 on the probability scale and do not entertain a priori implausible effect sizes (Banner et al., 2020) but also entertain substantially wider ranges of parameter values than we would have expected to observe prior to model fitting.

Models were fitted in Stan version 2.27 (Stan Development Team, 2021) via cmdstanr version 0.4.0.9000 and brms version 2.16.2. Models were run with four chains, each with 1000 warm‐up and 1000 sampling iterations per chain. Convergence diagnostics were monitored and models checked with posterior predictive checks. Estimates for all hyperparameters and fixed effects in the model are given in Appendix S1: Table S1. We also ran a model that had a structure similar to that of the detection‐occupancy framework but that modeled point‐occupancy directly. We found that this model generated the same broad conclusions as those reported in the main text, and these results are given in Appendix S2.

## RESULTS

**FIGURE 2:**
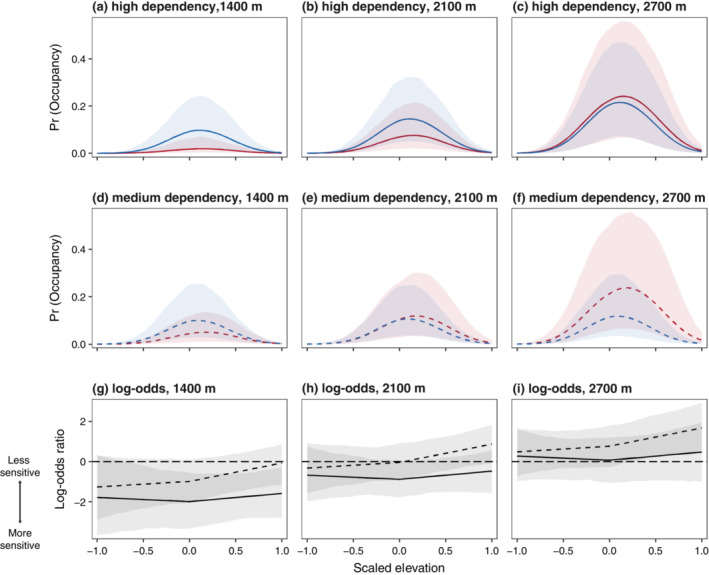
Elevational distributions for (a–c) high‐ and (d–f) medium‐forest‐dependency species found in different parts of elevational gradient and at varying levels of forest cover in surrounding landscape (red: 60%; blue: 100%). In each upper panel (a–f), lines indicate how a species' average occupancy probability, Pr(occupancy), varies with elevation and amount of forest in nearby landscape, with each panel representing an average species found in a different portion of the elevational gradient. The leftmost panels (a, d) represent high‐ and medium‐forest‐dependency species with an elevational range center of 1400 m above sea level (MASL), central panels (b, e) species with elevational range centers of 2100 MASL, and rightmost panels (c, f) species with elevational range centers of 2700 MASL (elevations range from 10th through 90th percentile of species' elevational midpoints). Lower panels (g–i) display log‐odds ratios (i.e., relative difference) between red and blue lines in panels above (solid line, high forest dependency; dashed line, medium forest dependency). Values <1 indicate reduced occupancy probabilities in landscapes with low forest cover, whereas values >1 indicate higher. Shaded areas represent 90% credible interval.

Across all species, elevational patterns of detection followed a unimodal distribution, with 93% (*n* = 2695) of point detections falling within published upper and lower range limits and the remaining 7% (*n* = 197) falling within the −2:2 interval (Appendix S1: Figure S5). In total, 69 species were detected outside of their published range bounds, with most of these detections falling within 400 m of the published elevational limits (*n* = 31; lower maximum = 578; upper maximum = 899), with the majority of these falling beyond the upper elevational limit (Mills, 2022d). At the species level, occupancy varied strongly by range position, with species displaying negative‐quadratic associations with elevation, producing bell‐shaped distributions with the peak of maximum occupancy tending to lie close to the elevational range midpoint (Figure 2).

### Interspecific gradients in sensitivity to habitat loss

**FIGURE 3:**
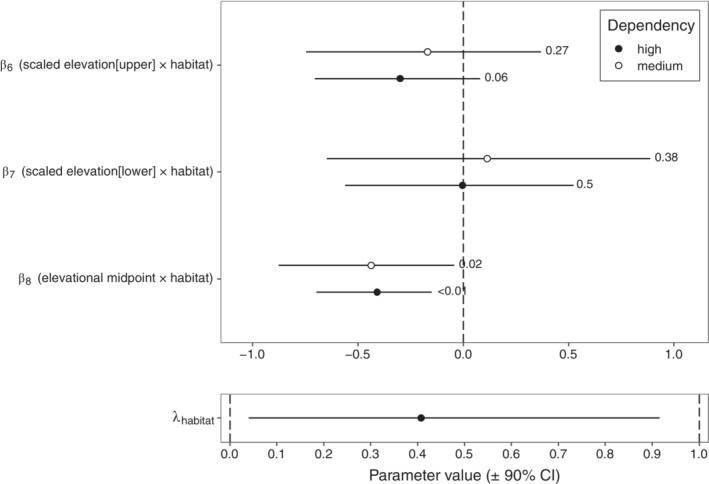
Model parameter values for focal terms relating to inter‐ and intraspecific patterns of elevational variation in sensitivity to forest loss. Upper panel: fixed effects for interactions between elevational range position and effect of forest loss (scaled elevation[upper] × habitat and scaled elevation[lower] × habitat) and the interaction between species' elevational midpoint and forest loss (elevational midpoint × habitat). Lower panel: phylogenetic signal in forest loss effect (λ_habitat_) (note that this scales between 0 and 1). Fixed effects are scaled to have unit SD, and parameter estimates are given with 95% credible interval (CI). The figures to the right of each CI in the upper panel (i.e., fixed effects) give the proportion of the posterior that lies in the opposite direction of the main effect (i.e., posterior probability that the direction of effect lies in the opposite direction from the point estimate).

**FIGURE 4:**
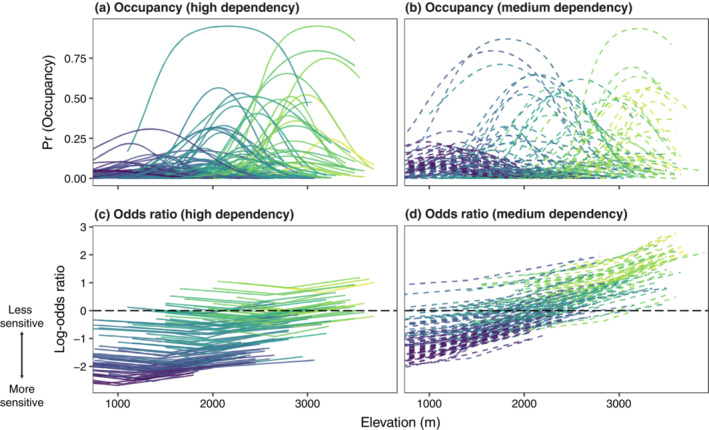
Elevational distributions for (a) high‐ and (b) medium‐forest‐dependency species at median forest cover (92% forest cover), with corresponding (c, d) species‐specific log‐odds ratios. As in Figure 2, the log‐odds capture the relative change in the odds of occupancy between deforested (60% forest cover) and intact (100% forest cover) landscapes. Curves are colored by species' elevational midpoints, ranging from blue (1000 m above sea level [MASL]) to yellow (3000 MASL).

For both medium‐ and high‐forest‐dependency species, there was marked variation in the effect of habitat loss across the elevational gradient (Figure 2). For high‐forest‐dependency species (Figure 2a–c), forest loss in the nearby landscape had large negative effects on occupancy toward lower elevations that weakened with increasing elevation, such that high‐elevation communities appeared relatively robust to loss of forest in the nearby landscape. The model was highly confident in the direction of this effect, with 0 lying outside the 95% credible interval (CI) and <1% of the posterior lying above 0 (i.e., probability of direction [PD]) (Figure 3). At the highest elevations, there were 12 high‐forest‐dependency species with slightly higher occupancies in landscapes with low forest cover (Figure 4a,c), though all had 90% CIs that strongly overlapped 0 (Appendix S1: Figure S6).

Medium‐forest‐dependency species (Figure 2d–f) were overall less negatively affected by loss of forest nearby (Figure 2g–i) and, by 2100 m, had occupancies in landscapes with low levels of forest cover equivalent to those with high forest cover. The model was confident in the direction of this effect, with 0 lying outside the 95% CI and 2% of the posterior lying above 0 (i.e., PD) (Figure 3). Toward upper elevations, occupancy is estimated to be slightly higher in landscapes with low forest cover (but note that the 90% interval substantially overlaps 0), with 58 species displaying higher log‐odds in landscapes with low forest cover (Figure 4b,d), two of which had a 90% interval that did not include 0 (Appendix S1: Figure S7).

Species were highly nonrandomly distributed across the elevational gradient with respect to phylogeny, with more closely related species tending to exist in more similar parts of the gradient than under random sampling (Phylogenetic Generalised Least Squares regressi of species' elevational midpoints: λ = 0.7; 95% CI: [0.24, 0.85]). However, although the effects of forest loss displayed some phylogenetic signal (λ = 0.41; 95% CI: [0.04, 0.92]; Figure 3), the large observed interspecific gradients appeared to arise independently of phylogeny.

### Intraspecific gradients in sensitivity to habitat loss

There was limited evidence of intraspecific sensitivity to deforestation across elevational gradients for either high‐ or medium‐forest‐dependency species (Figure 2). CIs for the coefficients determining sensitivity toward lower range margins strongly overlap 0 for both high‐ (PD = 38%) and medium‐dependency (PD = 50%) species. Effects were slightly larger toward upper range margins, though still fairly marginal, with 95% CIs for the coefficients determining sensitivity toward lower range margins again overlapping 0 for both high‐ (PD = 27%) and medium‐dependency (PD = 6%) species. Effects on the difference in occupancy probability are marginal, with only minor differences in the elevational distributions of species between high and low forested landscapes (Figure 2).

## DISCUSSION

Community‐level responses to habitat conversion displayed strong elevational dependencies, but how these were underpinned by variation in sensitivity at the species and population levels was unclear (Orme et al., 2019; Peters et al., 2019). Across 243 species with some degree of forest dependency, we found good evidence of interspecific elevational gradients in sensitivity to loss of forest in the nearby landscape, with species toward lower elevations substantially more impacted than those at higher elevations, an effect that arises independently of phylogeny. Conversely, we find limited evidence of intraspecific elevational gradients in sensitivity, with sensitivities varying little across species' elevational ranges. Elevational gradients in the biodiversity response to land‐use change in the montane tropics (Peters et al., 2019) thus appear to be underpinned by variation across elevation in sensitivity of species to habitat loss, rather than as a consequence of gradients of predisturbance species richness (Quintero & Jetz, 2018) or population proximity to climatically limiting conditions lower on the mountain or at range margins. Although the negative effects of forest removal in the nearby landscape are well known (e.g., Amazonia, Barlow et al., 2016; Socolar et al., 2019), our results represent the first empirical demonstration of variation in the strength of these effects along an elevational gradient.

### Interspecific gradients in sensitivity to habitat loss

We uncovered strong interspecific elevational gradients in sensitivity, with species low on the elevational gradient tending to exhibit significantly higher log‐odds of occupancy in intact versus degraded landscapes, whereas many species at the highest elevations appeared relatively unaffected by forest loss in the nearby landscape. Toward the highest elevations, some medium‐dependency forest species exhibited higher occupancies in landscapes that did not have high levels of forest cover, suggesting that they might be performing even better in patchily forested landscapes that contain high levels of forest edge and scrub. These results are broadly consistent with the idea that biogeographic traits involving species ranges and habitat associations are key predictors of disturbance sensitivity in birds (Socolar & Wilcove, 2019).

One possible explanation for this result is that elevational variation in forest structure and configuration may have resulted in a high‐elevation bird community that is preadapted or filtered for species that can survive when levels of forest cover in the landscape are low (Betts et al., 2019). At higher elevations, forests take longer to recover following natural disturbance (Guariguata, 1990) and exhibit structural differences, such as reduced stature, loss of vertical stratification (Terborgh, 1977), and a more open canopy (Asner et al., 2014). Forest in these high‐elevation landscapes is sparsely distributed, ultimately becoming interspersed with stunted vegetation and grassland toward the treeline. These natural gradients in forest structure and configuration have been heightened by the long history of human habitation and significant forest removal in the high Andes (Sylvester et al., 2017). Indeed, across much of the upper‐elevational gradient of the Andes, contemporary levels of forest cover, though low (Armenteras et al., 2003), could represent a gain in total forest extent since the pre‐Columbian era (Åkesson et al., 2020).

Systematic differences in forest structure and configuration across the elevational gradient suggest that toward higher elevations there may have been a persistent selection pressure for ability to continue in landscapes that contain many of the features associated with disturbances inherent in forest loss at lower elevations (i.e., loss of structural complexity, reduced canopy cover, and fragmentation). Though the factors that underpin the reduced sensitivity of high‐elevation birds are unclear, they may relate to the low gap‐crossing propensity of lowland birds (Lees & Peres, 2009) or an inability to tolerate high levels of ambient light (Ausprey et al., 2021). Although we are unaware of studies that examine gap‐crossing or dispersal ability across elevational gradients, morphological traits associated with dispersal can vary markedly across environmental gradients. For example, the hand‐wing index, a proxy for dispersal ability, increases significantly over temperature and precipitation gradients, as well as by latitude (Sheard et al., 2020), suggesting potential for systematic differences in gap‐crossing propensity between low‐ and high‐elevation avifaunas (Neate‐Clegg et al., 2021).

The high sensitivity of low‐elevation forest avifauna to forest loss may be further exacerbated by elevational gradients in the ecological specialization of the forest community. Previous work along an Andean elevational gradient indicated that lower‐elevation bird communities might be more ecologically specialized, with narrower ecological niches (Pigot et al., 2016). Ecological specialization may arise through highly specific microhabitat tolerances or specialized dietary regimes that predispose species to respond poorly to landscape‐scale forest degradation (Curtis et al., 2021). Though identifying specific life histories that may contribute to the observed elevational gradient in sensitivity were beyond the scope of this study, further exploration of these more proximate factors constitutes a direction for future work.

### Intraspecific gradients in sensitivity to habitat loss

We found limited evidence of heightened sensitivity to forest loss toward either lower elevations or toward species' elevational range margins, suggesting that habitat features that influence a species' probability of occupancy are equally important, regardless of where a population exists on the elevational gradient. This contrasts with theory that suggests that, owing to proximity to regions of climatic limitation (e.g., thermal tolerance thresholds; Sunday et al., 2014), the effects of habitat loss will be relatively more severe toward low elevations, where temperatures are highest (Peters et al., 2019), or toward species range margins, where populations are less common, less abundant, and potentially closer to species‐specific climatic thresholds (Lee‐Yaw et al., 2016; Orme et al., 2019).

One potential explanation for this is that species’ elevational distributions are, on average, only weakly related to climate, with nonclimatic factors, such as competitive exclusion (Jankowski et al., 2010) and habitat structure (Terborgh, 1977), being relatively more important for determining species range margins. Although species’ range margins often represent regions of reduced climatic suitability (Lee‐Yaw et al., 2016), the extent to which this applies to species’ elevational range limits is unclear. Previous work found, for example, that species' elevational distributions of tropical birds were poorly explained by the thermal limits or energetic costs of thermoregulation (Freeman, 2016; Londoño et al., 2017). Conversely, upslope shifts and abundance changes in tropical birds in response to climate change are consistent with elevational distributions being at least partially constrained by temperature, potentially via interactions with habitat or biotic interactions with prey (Freeman et al., 2018). These conflicting results suggest that associations between species' elevational distributions and climate are likely to be complex and potentially case‐specific, depending on the species and region in question (Elsen et al., 2017), weakening many of the arguments for heightened sensitivity to elevational range margins.

Alternatively, forest extent and configuration (both indexed by total forest cover in the landscape) may be relatively limited in their ability to ameliorate declining climatic suitability toward range margins. Habitat features that are involved in regulating population response to climatic variation may act at very fine spatial scales (González‐del‐Pliego et al., 2020) that are weakly related to forest characteristics at the landscape scale (Senior et al., 2017, 2018). As a consequence, some habitat features, not assessed in our study, may act to buffer populations from climatic variation at range margins. Outside of the tropics, fine‐scale microclimatic variation has been related to population persistence in anthropogenically modified landscapes (Suggitt et al., 2018), and future work should consider how other habitat features can influence population persistence toward range margins.

### Conservation implications and conclusions

Our results demonstrate strong elevational dependencies in the indirect effects of habitat loss on forest avifauna of tropical mountains. Elevational gradients in community‐level sensitivity to land‐use change appear to be underpinned by the heightened sensitivity of species found at low elevations, as opposed to varying sensitivity of populations present in different positions along a gradient or through elevational gradients in predisturbance species richness. The limited intraspecific effect further suggests that elevational gradients in biodiversity response are not related to climatic thresholds per se but are better understood in terms of the sensitivity of species that make up the community found at different elevations.

Further work is needed to integrate these patterns of sensitivity with species range sizes and patterns of endemism to develop a holistic set of conservation recommendations for these regions. Across much of the tropics, including within Colombia, the proportion of land that is protected declines toward lower elevations (Elsen et al., 2018; Forero‐Medina & Joppa, 2010), coinciding with increasing levels of human pressure (Elsen et al., 2020). Some of the highest rates of contemporary deforestation can be found at lower elevations, such as along the foot of the Andes (e.g., Caqueta deforestation frontier; Murad & Pearse, 2018), and our results suggest that biodiversity outcomes may be particularly severe in these regions. In addition to harboring a highly sensitive avifauna, forests that exist toward mountain peripheries also provide critical pathways for species to move upslope and thereby maintain their thermal niche in response to rising temperatures (Brooks et al., 1999; Lawton et al., 2001). Protection of these forests is therefore particularly crucial to mitigate biodiversity losses in the face of climate change.

## CONFLICT OF INTEREST

The authors declare no conflict of interest.

## Supporting information


Appendix S1

Appendix S2

## Data Availability

Elevation and range data (Mills, 2022d) are available in Figshare at https://doi.org/10.6084/m9.figshare.19745377.v2. Bird detection data and all elevation and habitat covariates (Mills, 2022a) are available in Figshare at https://doi.org/10.6084/m9.figshare.19741999.v1. The consensus phylogeny used in the detection‐occupancy model (Mills, 2022c) is available in Figshare at https://doi.org/10.6084/m9.figshare.19742044.v1. Code for analyses and data set formatting (Mills, 2022b) is available in Figshare at https://doi.org/10.6084/m9.figshare.20424603.v1.
