# Supplementary material for: High sensitivity of tropical forest birds to deforestation at lower altitudes
Source: Ecology. 2022 Nov 14;104(1):e3867. doi: 10.1002/ecy.3867 (PMC10078351; doi:10.1002/ecy.3867)
Supplement: Supplementary file 1 — Appendix S1 [file ECY-104-0-s002.pdf]

Appendix S1

Title: High sensitivity of tropical forest birds to deforestation at lower altitudes

Authors: Simon C. Mills, Jacob B. Socolar, Felicity A. Edwards, Edicson Parra, Diego E. Martínez-Revelo, Jose Manuel Ochoa Quintero, Torbjørn Haugaasen, Robert P. Freckleton, Jos Barlow, David P. Edwards

Journal: Ecology

Table S1 Model summary table with parameter estimates for hyperparameters and fixed effects.

Class	Name	Group	Mean	Lower CI	Upper CI
Occupancy fixed effect	Intercept		-2.51	-3.39	-1.05
	range_pos		**1.28**	**0.71**	**1.86**
	range_pos2		-4.97	-5.68	-4.29
	ele_midpoint		**0.64**	**0.15**	**1.14**
	habitat		**0.33**	-0.11	**0.79**
	medium_dependency		-0.03	-0.47	**0.38**
	range_size_sc		**0.21**	-0.23	**0.65**
	ele_midpoint:habitat		-0.41	-0.70	-0.14
	range_pos:habitat		**0.00**	-0.55	**0.54**
	habitat:range_pos_upr		-0.31	-1.06	**0.47**
	range_pos:medium_dependency		**0.04**	-0.43	**0.52**
	range_pos2:medium_dependency		-0.11	-0.55	**0.33**
	ele_midpoint:medium_dependency		**0.24**	-0.22	**0.69**
	habitat:medium_dependency		**0.20**	-0.05	**0.45**
	habitat:range_size_sc		-0.05	-0.26	**0.16**
	ele_midpoint:habitat:medium_dependency		-0.03	-0.28	**0.22**
	range_pos:habitat:medium_dependency		**0.12**	-0.33	**0.57**
	habitat:medium_dependency:range_pos_upr		0.00	-0.58	**0.56**
Occupancy random effect	sd_intercept_species		**2.21**	**1.78**	**2.69**
	sd_habitat_species		**0.43**	**0.04**	**0.72**
	sd_range_pos_species		**2.87**	**2.30**	**3.53**
	sd_range_pos2_species		**2.04**	**1.52**	**2.62**
	sd_intercept_cl_sp		**2.30**	**2.00**	**2.62**
	sd_intercept_site_sp		**3.00**	**2.61**	**3.45**
	sd_intercept_phylo		**0.65**	**0.04**	**1.93**
	sd_habitat_phylo		**0.29**	**0.02**	**0.77**
	cor_phylo	intercept, habitat	-0.18	-0.98	**0.92**
Detection fixed effect	Intercept		-2.37	-2.74	-2.00
	time		-0.27	-0.34	-0.20
	obsvrJS		**0.06**	-0.22	**0.35**
	obsvrSCM		**0.31**	**0.01**	**0.62**
Detection random effect	sd_intercept_obs_sp		**0.52**	**0.39**	**0.67**
	sd_intercept_site		**0.21**	**0.08**	**0.40**
	sd_intercept_species		**1.12**	**0.94**	**1.33**
	sd_time_species		**0.34**	**0.27**	**0.42**
	cor_species	intercept, time	-0.28	-0.51	-0.02

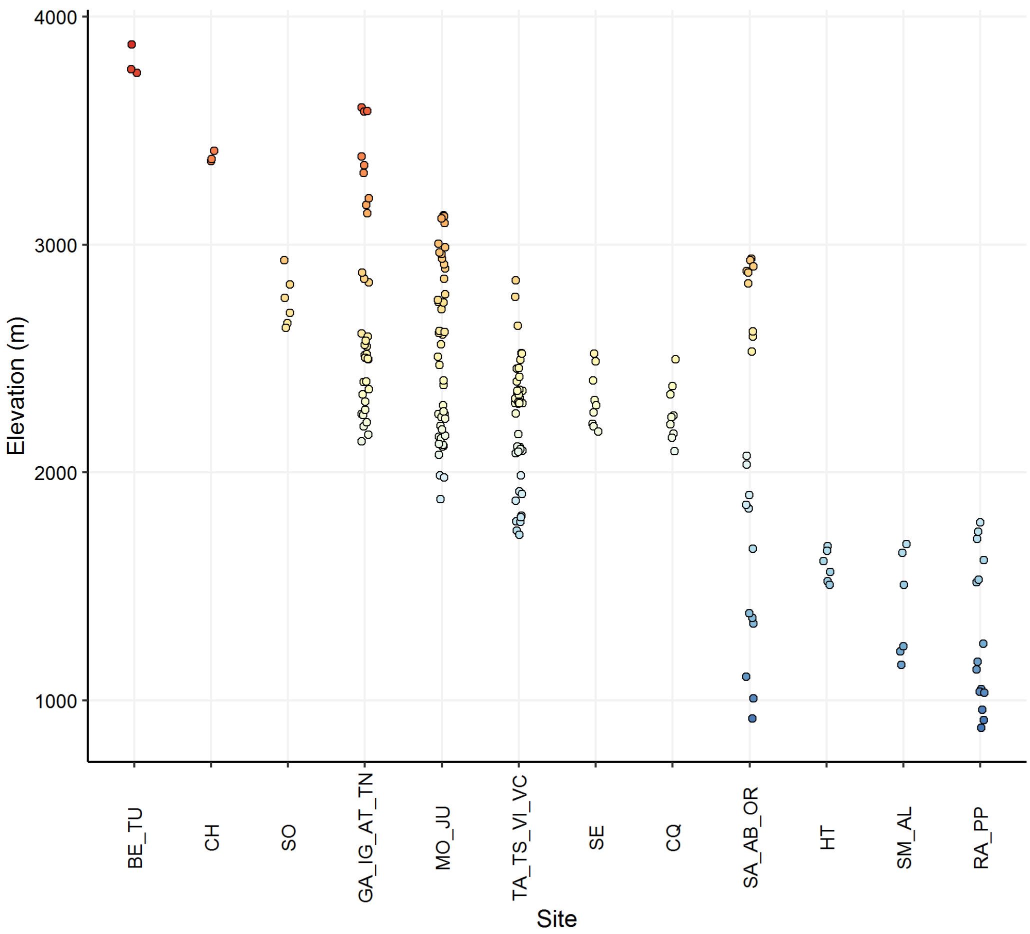

Figure S1 Point elevations, split by site and ordered by site median elevation. Points have been jittered along the horizontal axis to prevent overplotting.

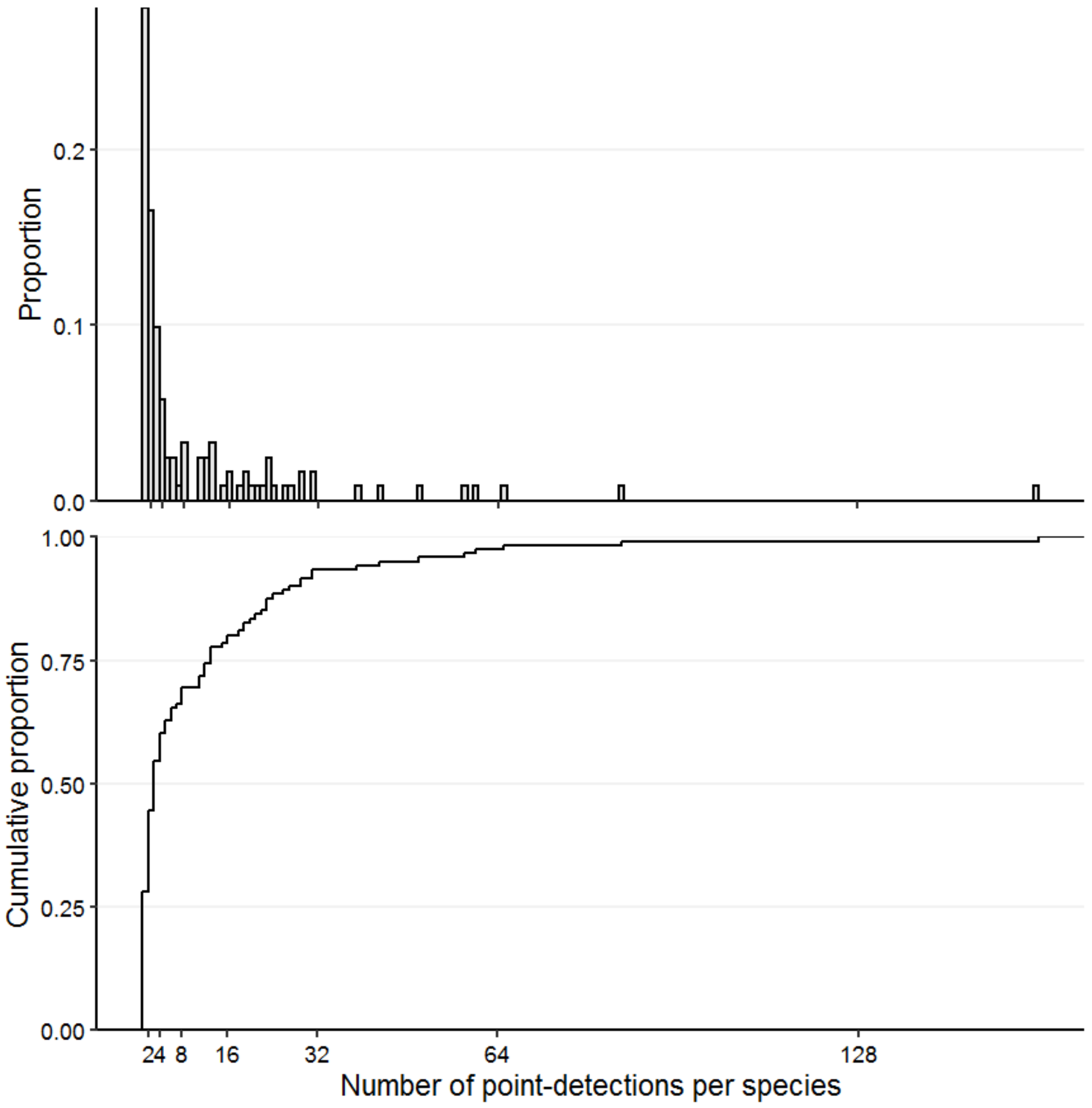

Figure S2 Distributions of point-level detections by species (upper panel), with this same information displayed cumulatively in the lower panel. 30% of the 191 species were detected on 1 point, 43% were detected on ≤ 2 points, 52% were detected on ≤ 3 points; 24% species were detected on ≥ 10 points.

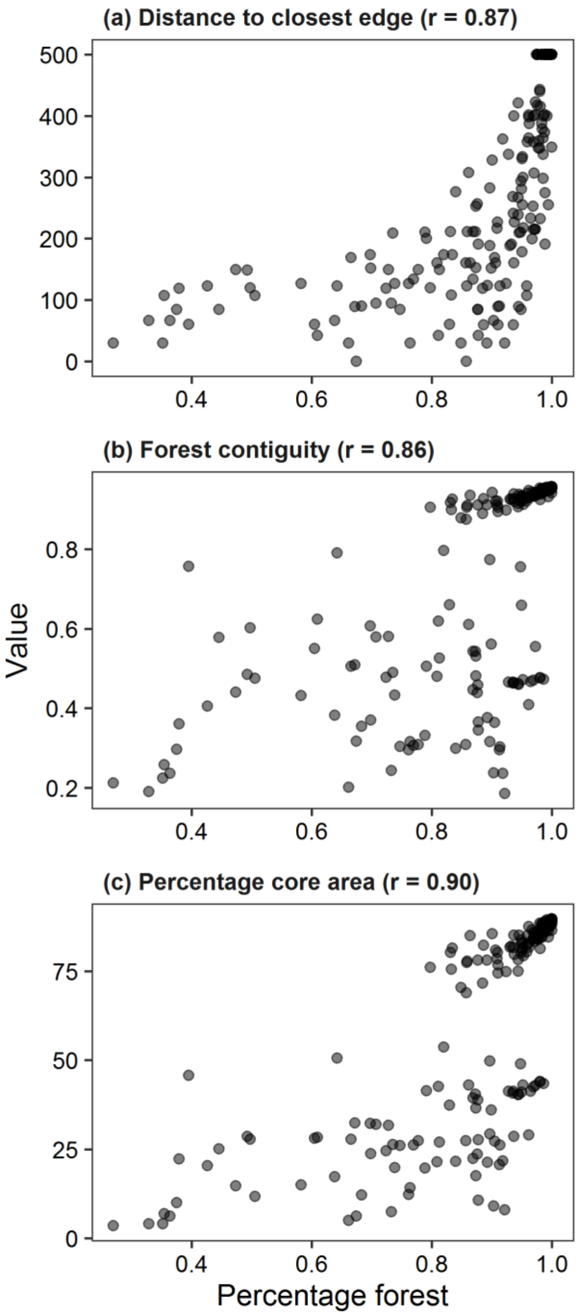

Figure S3 Covariation in forest cover and configuration metrics: (a) area of forest edge, defined as area of forested pixels that are not adjacent to non-forest pixels (b) distance to closest edge, defined as the distance from the point to cells with less than 50% forest cover, (c) forest

contiguity, an index of how interspersed forest cells are with non-forest cells, and (d) percentage core area, the percentage of forested cells that are not adjacent to non-forest cells.

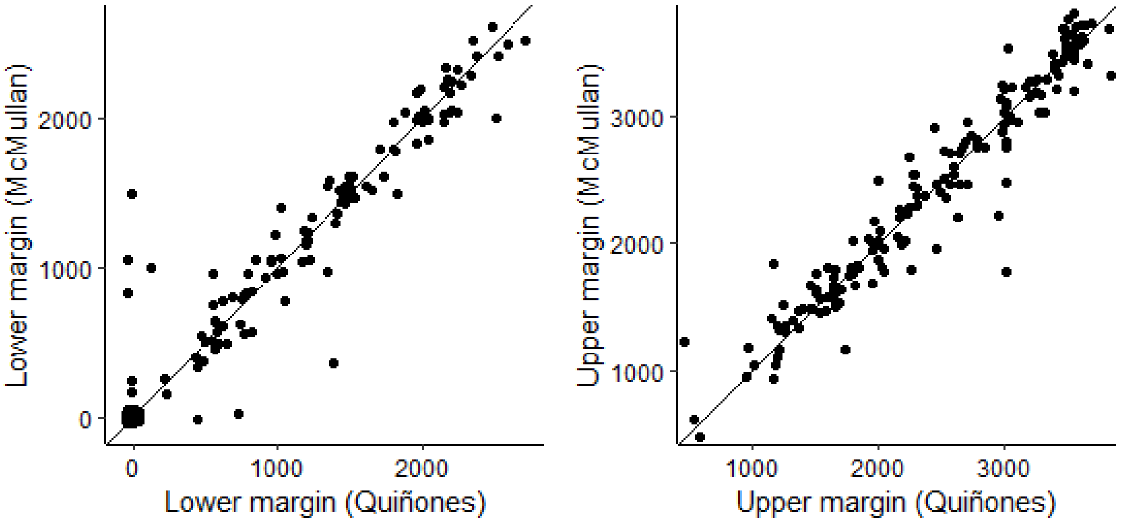

Figure S4 Upper and lower range margins from Ayerbe Quiñones and McMullan. To avoid overplotting, points have been jittered +- 50m along the horizontal and vertical axes.

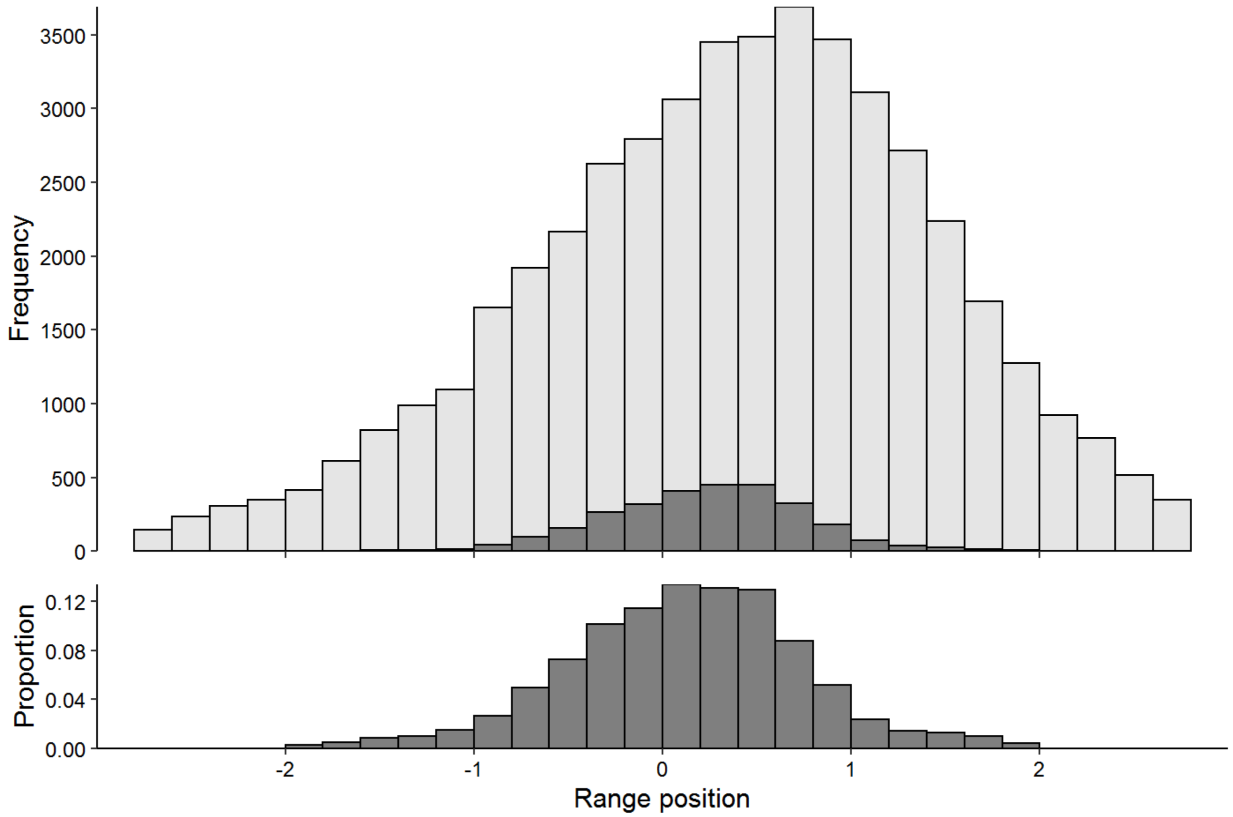

Figure S5 Distribution of detections (dark grey) and non-detections (light grey) across all species × point combinations (note that data were clipped at ±3 units of scaled elevation to avoid modelling the large numbers of all-0 detection histories that exist far away from a species' range). Upper panel has the total numbers at each range position, while lower contains the same information but expressed as the proportion of species × point combinations that are detections in each elevational bin.

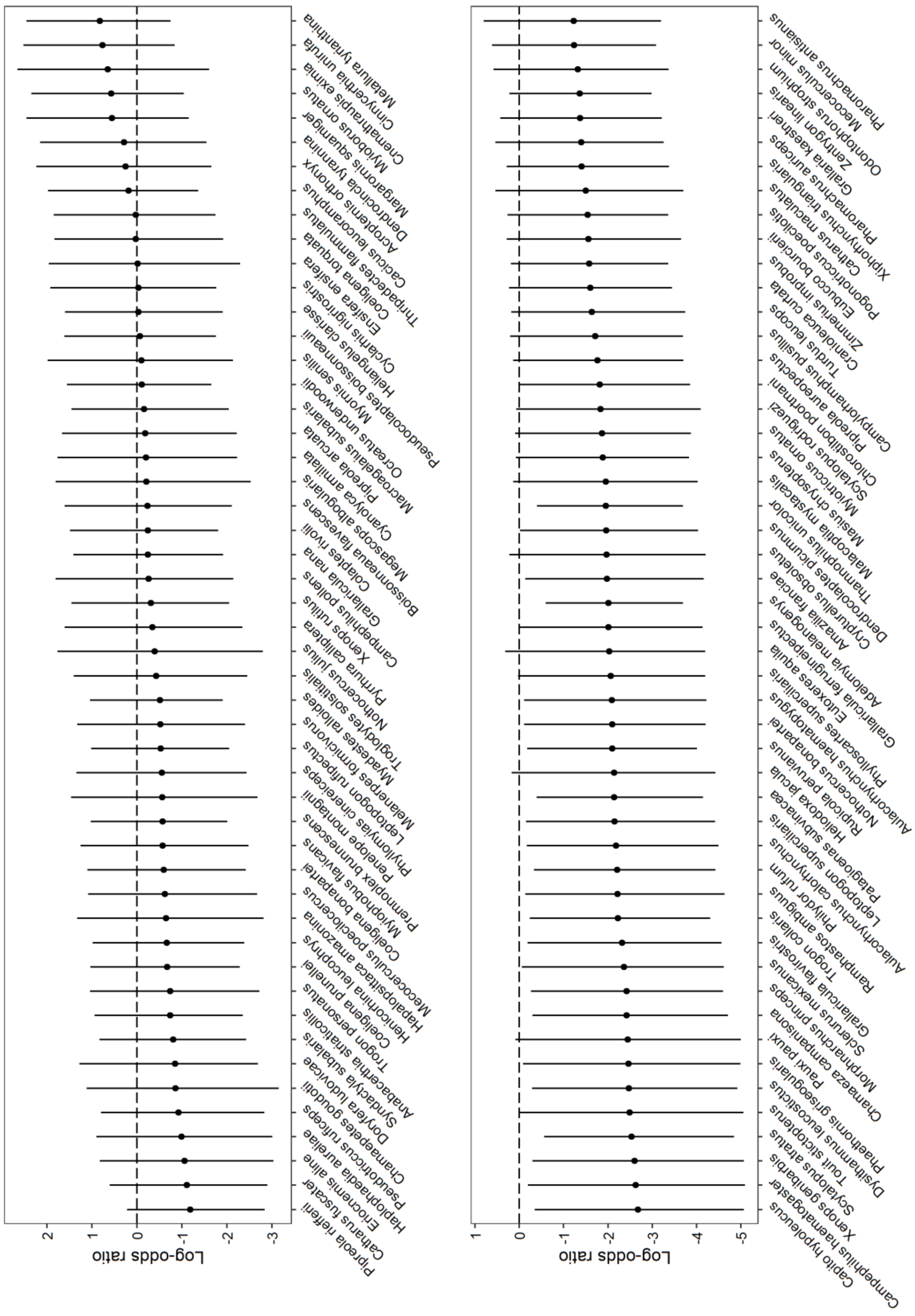
Log-odds ratio
Log-odds ratio

Figure S6 Log-odds ratios between 90th and 10th percentile forest cover for each species' at their range centre for high forest-dependency species, with bars representing the 90% credible interval.

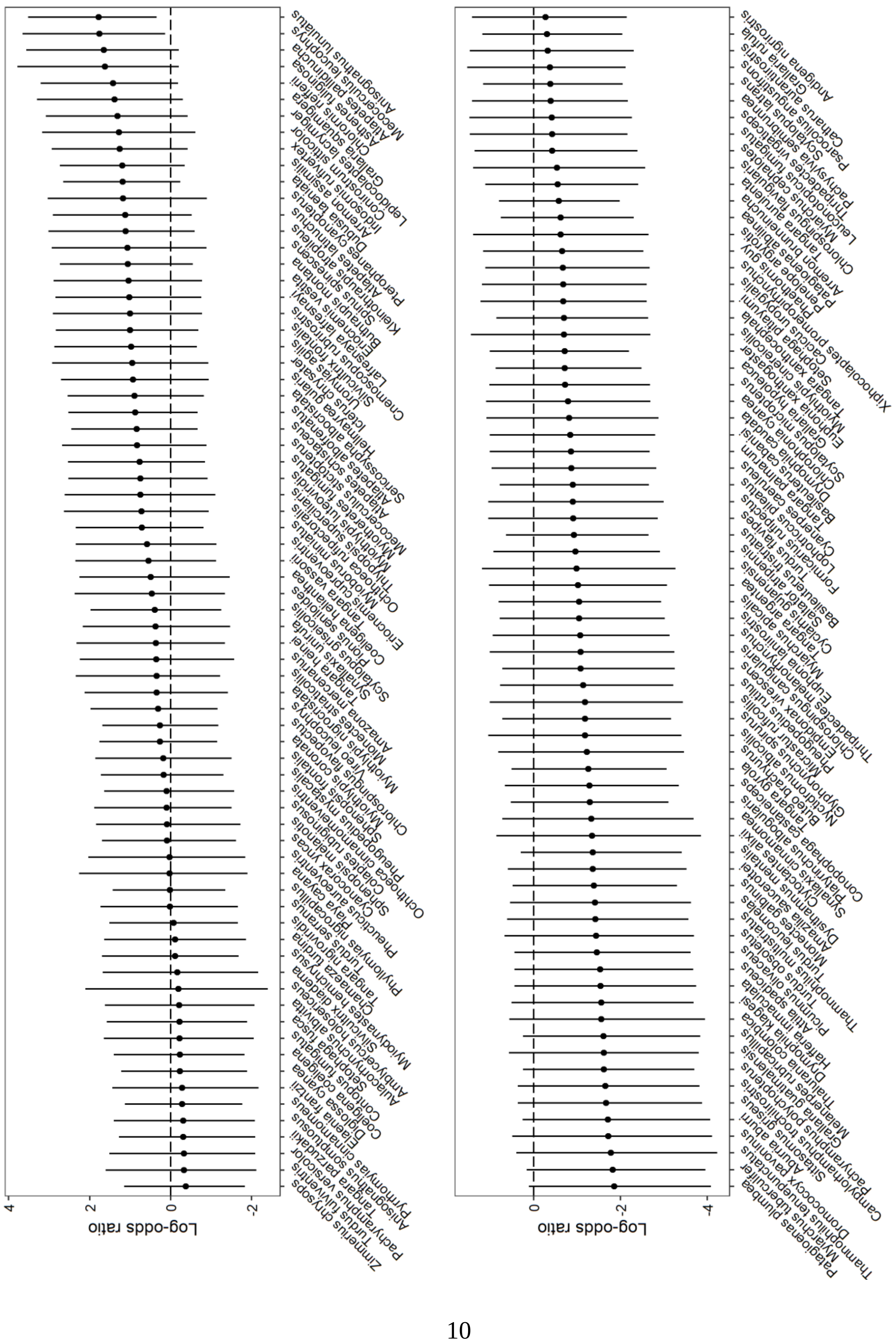

Log-odds ratio
4
2
0
-2
Log-odds ratio
0
-2
-4

Figure S7 Log-odds ratios between 90th and 10th percentile forest cover for each species' at their range centre for medium forest-dependency species, with bars representing the 90% credible interval.
