# Supplementary material for: High sensitivity of tropical forest birds to deforestation at lower altitudes
Source: Ecology. 2022 Nov 14;104(1):e3867. doi: 10.1002/ecy.3867 (PMC10078351; doi:10.1002/ecy.3867)
Supplement: Supplementary file 2 — Appendix S2 [file ECY-104-0-s001.pdf]

Appendix S2

Title: High sensitivity of tropical forest birds to deforestation at lower altitudes

Authors: Simon C. Mills, Jacob B. Socolar, Felicity A. Edwards, Edicson Parra, Diego E. Martínez-Revelo, Jose Manuel Ochoa Quintero, Torbjørn Haugaasen, Robert P. Freckleton, Jos Barlow, David P. Edwards

Journal: Ecology

We additionally ran a model that does not use the detection-occupancy modelling framework, and instead is a simpler logistic regression of point-level detection. This model retains the same broad structure as the main model presented in the *Statistical Analysis* section but does make use of the information obtained from replicated sampling and instead just models point-level detection directly. Model structure is:

$$
\begin{aligned}
Q_{ik} = {} & u_{cluster[i],k} + \gamma_{site[i],k} + \beta_{0,k,dep[k]} + \beta_{1,k,dep[k]}\text{scaled elevation}_{\text{i,k}} + \beta_{2,k,dep[k]}\text{scaled elevation}^2_{i,k} \\
& + \beta_{3,dep[k]}\text{elevational midpoint}_{\text{k}} + \beta_{4,k,dep[k]}\text{habitat}_i \\
& + \beta_{5,dep[k]}\text{elevational midpoint}_k.\text{habitat}_i + \beta_{6,dep[k],rhalf[i]}\text{scaled elevation}_{i,k}.\text{habitat}_i \\
& + \beta_{7,dep[k]}.\text{range breadth}_k + \beta_{8,dep[k]}.\text{range breadth}_k.\text{habitat}_{\text{i}}
\end{aligned}
$$

The definition of coefficients and covariates follow those given in the main text. The key difference between this model and the one presented in the main text is that now, rather than modelling $logit(\psi_{ik})$, or the probability of occupancy for the *i*th species on the *k*th point, we instead model the raw point-level observations, Q_{ik}, directly, and are therefore unable to separate factors that affect occupancy from those that affect detection.

This latter model returns values for our focal coefficients that support the same conclusions as we draw from the main model, with the same direction of effect and overlap (or lack thereof) of CIs with 0 (Appendix S2: Figure S1).

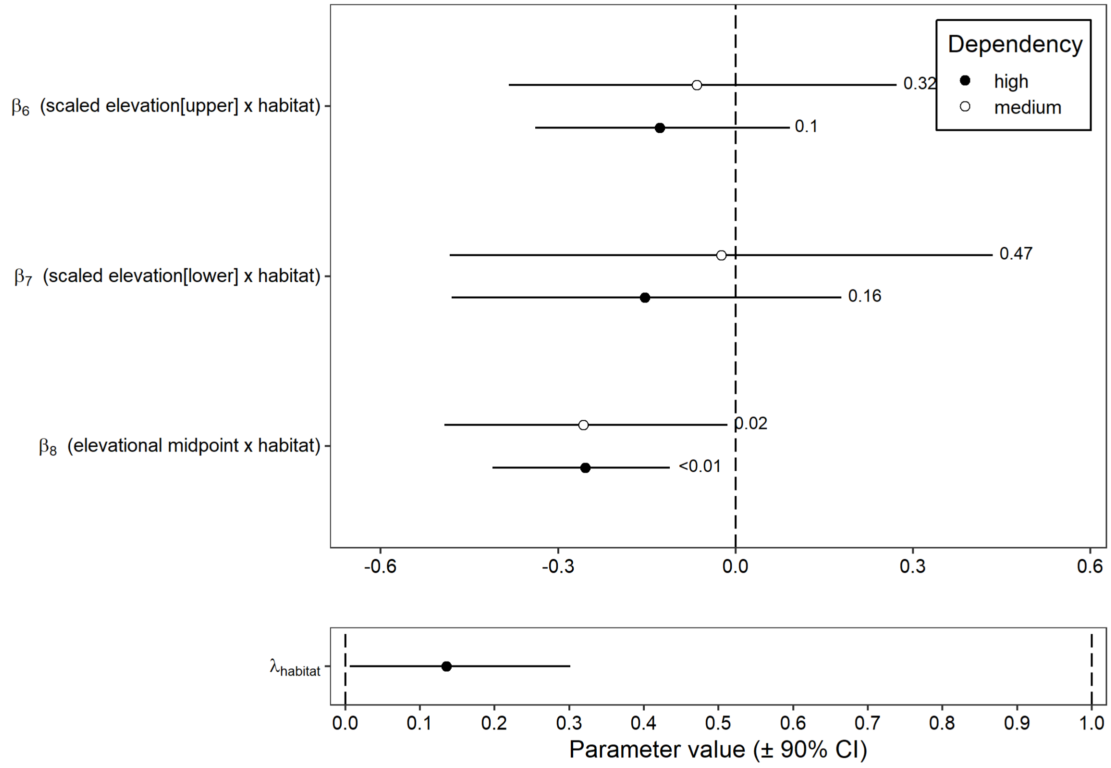

Figure S1 Model parameter values for focal terms relating to inter- and intra-specific patterns of elevational variation in sensitivity to forest loss, for the model fitted to raw point-level observations. The upper panel displays the fixed effects for the interactions between elevational range position and the effect of forest loss (scaled elevation[upper] × habitat and scaled elevation[lower] × habitat), and the interaction between species' elevational midpoint and forest loss (elevational midpoint × habitat). The lower panel displays the phylogenetic signal in the forest loss effect ($\lambda_{habitat}$): note that this scales between 0 and 1. Fixed effects are scaled to have

unit standard deviation and parameter estimates are given with 95% credible interval (CI). The figures to the right of each CI in the upper panel (i.e. fixed effects) give the proportion of the posterior that lies in the opposite direction of the main effect (i.e., posterior probability that the direction of effect lies in the opposite direction from the point estimate).
